# Obstetrics and Gynæology

**Published:** 1877-05

**Authors:** 


### Summary of Progress in the Medical Sciences.

### I. Obstetrics and Gynecology.

*Viability of a 6 months fœtus.* Dr. Githens. (*Amer. Jour of Obstet. and Diseases of Women and Children.* Oct., 1876.

The child was born May 20th, 1876. Dr. G. would not allow it to be washed, and had it wrapped in cotton, which was changed daily. This dressing was continued for two or three weeks. The child was fed on milk and cod's liver oil until able to nurse. "This infant has shown its ability to support an independent existence at the early age of 178 days, or six calendar months," a conclusion reached by Dr. G. after carefully determining the exact time of the last visit home of the father of the child.

*Synopsis of Four Hundred Cases of Obstetrical Practice.* Kinsman. (*The Ohio Med. Record*, Feb., 1877.)

Of the four hundred cases of labor that occurred among well-to-do people, there were born, of three hundred and forty-seven mothers, four hundred and eleven children. Of the cases, nine were twins, and one triplets. Some part of the cephalic extremity presented in three hundred and eighty-nine cases; and of the face, two. Back presentations, six, one of knee and four transverse.

There were twenty-five deliveries with forceps; of these, it was applied three times at superior strait. Podalic version was performed five times. "Cephalic version" was not successfully performed, the waters escaping being the cause of failure. Of the five children turned, three were born living, the other two were dead before operation.

Only one case of prolapsus of funis is mentioned. One case of short cord, 10 inches in length, still birth, is reported; eleven were born dead; five had been dead for some time, one perished in placenta prævia, one in rupture of uterus, one from cerebral hemorrhage, one footling, death occurring, after escape of body, two while mothers were in convulsions, and one delivered dead with forceps. Six mothers died from the immediate cause of labor, viz.; Rupture of uterus, placenta prævia, puerperal convulsions, embolism of middle cerebral artery, septicæmia and peritonitis. The complications of labor were as follows: Convulsions, four times; agglutination of neck of womb, once; rupture of uterus, once; fibroid tumor, once; placenta prævia, three times; adherent placenta, twice; post-partum hemorrhage, ten times.

Of the defects of organization, the following cases were reported: One of imperforate anus, one of atresia of vagina, one of agglutination of meatus urinarius, one of hair lip, one of redundant fingers and toes, and one of cyanosis from cardiac defect. Of the three hundred and forty-seven cases, one hundred and nine were primiparæ. The second stage of labor averaged about four hours. The longest labor was seventy-two hours, and occurred in a woman forty-seven years of age. The forceps were used in twenty-five cases; the following occasioned its use: convulsions, three times; impacted head, three times; spasms of uterus, five times; face presentation, once; exhaustion, with head at sup.strait, three times; threatened exhaustion, with head in sup.strait, eight times; failure to rotate, two times.

Of the forceps deliveries, four children were born dead, two where the mother had convulsions, one where there was threatened peritonitis, and one impaction of the head. Two of the mothers that were delivered with forceps died; of septicaeima, one, and of peritonitis, one.

W. F. L.

*The Relations of Albuminuria to Pregnancy.* MARTIN. (*Proceedings of the Med. Society of the County of Kings.* Vol. I. No. 10.)

That albuminuria in pregnancy is vital and not mechanical, is argued, and supported by a collection of cases by Dr. M. The cases reported show that; death of the ovum will relieve severe and progressive uremic symptoms, even without its removal from the uterus. One of the cases is as follows: A lady, having passed a single period only, consulted her physician for relief from distressing headache, disturbances of vision, nausea, etc. Albumen having been discovered in the urine, the usual remedies were applied, without benefit. Interference with the impregnated uterus being denied her, she went to a quack doctor in New York City, who introduced a sound into the uterus every week, for seven consecutive weeks before the contents were discharged.

Patient said that soon after the first introduction of sound, she experienced great relief. Returning home to her family doctor, her urine was examined, and found perfectly normal. Two years later she again became pregnant, the same line of symptoms following as before, and the urine loaded with albumen. Again the ovum was destroyed, with same relief to patient. Ten days after the uterus had been emptied, urine was examined, but showed no trace of albumen. In the cases reported, no cause coincident and concurrent with pregnancy, and yet independent of it could be detected. As to the origin of albuminuria in early pregnancy, when the womb is not sufficiently large to make pressure on the renal circulation, the author says: "When an ovum is fertilized, a profound impression must be made upon the nervous centres, which preside over the process of nutrition. The rapid and complete growth of the fœtus; the establishment of a new vascular system for its support, demand large applications of nutritive force. The medium through which these impulses are transformed into actions, is the great sympathetic nerve. The first steps toward any change of nutrition are accomplished by the agency of the vaso-motor nerves. The blood being ready to furnish material, the capillary circulation must be ready to take it up. And this condition of excitation must be maintained by the action of the sympathetic nerves during the whole time that an extra supply is needed. The uterus and kidneys are certainly associated organs, and it is, therefore, at least probable, that an influence derived from the unusual nutritive activity in the uterus may be reflected from the nervous centre through the usual nerves, and being continuous, may stimulate or alter the interstitial circulation of the kidney in such a way as to produce albuminuria."

W. F. L.

*Child-bearing and its Effects on certain forms of ear disease.* F. M. PIERCE, M. D., of Manchester. (*Obstet. Jour. of Great Britain and Ireland*, 1876.)

P., in a paper read before the last meeting of the British Medical Association, drew attention to the occurrence amongst a certain class of patients, of marked increase in deafness, and in the gravity of the symptoms of ear disease, due to pregnancy, parturition, &c. The form of aural mischief most aggravated by these processes, was chronic non-suppurative inflammation of the tympanic cavities. After each confinement, the patients were much worse, the hearing diminished, and the tinnitus aurium was more marked. The deterioration was very persistent, and extremely obstinate; and ultimately, after repeated confinements, the hearing was almost entirely abolished. Young, strong, and apparently healthy females were the chief sufferers; often they had never had any ailment in their lives. The aural deterioration began with pregnancy, and increased onwards to parturition, after which the effect remained: a result by no means comparable with the temporary aggravation seen during other constitutional affections, fevers, &c. Other forms of ear disease were not affected in the same permanent manner as chronic non-supperative inflammation of the tympanic cavities. No history of any syphilitic taint could be detected in these cases. Whether the effect on the aural condition produced by child-bearing was only part of a general diminution of nerve power, and in no way due to the special condition of pregnancy, &c., apart from its constitutional deterioration, was matter for further observation, though the facts were in favor of its being caused by the state peculiar to pregnancy. Early attention to treatment was most important to these patients.

*Premature labor, induction of, by a new apparatus.* DR. CHASSAGNY (*Archives de Tocologie*, 1876.)

Dr. Chassagny describes a new apparatus for the induction of premature labor. It consists of a double dilating bag, each bag being filled by a separate tube, so that the tube which communicates with the upper bag passes through the lower. The lower bag is made of thick india-rubber. It is inserted into the vagina, and dilated with air or water, the effect of which is to induce uterine contractions. The upper bag, which is made of extremely thin india-rubber, is then filled with water. As it is supported by the thick bag below, the effect of this is that it insinuates itself into every pouch of the vagina, and sends out a finger-like projection into the cervix, which, as the author believes, effects its dilatation with far greater mechanical advantage than even the natural pouch of liquor amnii. By experiments on models made to represent the vagina and cervix, he has satisfied himself that this projection of a finger-like pouch into the cervix does actually take place, even when the cervix forms at first a prominence, and the os is undilated. He therefore considers that his double dilating bag is far superior to the dilating bag of Gariel, or the colpeurynter of Braun, which merely distends the vagina, and often brings on active labor pains only after a long interval. It is also far easier of application than intra-uterine dilators, which require to be frequently reinserted, and it can be used even when there is, at the commencement, no dilatation whatever of the os. The methods of puncturing the membranes, of dilating the os with tents, or of introducing an elastic bougie, the author considers to be exploded as a means of inducing labor.

A case is related in which the method was successfully applied. The patient, aged forty-seven, was reduced to the last extremity by uncontrollable vomiting, and induction of labor was commenced about three weeks before full time. The os was extremely high up, hard, rigid, and nodular, and would not admit even the point of the finger. Pains were brought on soon after the introduction of the bag, the os was dilated to the size of a franc after five hours, and labor was completed after about twenty-four hours, although on two occasions the bags were removed for some hours, during which time pains entirely ceased, and the os became again contracted. This cessation of pain appeared to be due to the exhausted state of the patient.

The application of the double dilating bag is not confined to the induction of labor, but, in the author's practice, it supersedes all other means in many cases. Thus it is used instead of tents to dilate the cervix of the unimpregnated uterus, in order to explore its cavity, to restrain hæmorrhage, to remove polypi, or to make intra-uterine applications. In cases of placenta prævia it effects a complete dilatation of the cervix without allowing the slightest hæmorrhage to continue, an effect which the author has observed in a dozen cases. When the insertion completely covers the os, he pierces the placenta through the centre, and finds that the bag insinuates itself into this opening with an equally good result. But it is in post-partum hæmorrhage that the results are most striking. The uterus is first emptied of all clots, and the bags are then introduced and dilated with water. The thin bag then insinuates itself into the uterus, and completely fills it, compressing the open mouths of all the bleeding vessels. When the uterus begins to contract, it may be allowed to expel the water, the open mouth of the tube being kept at a high level. There is then perfect security that no cavity is formed into which hæmorrhage could take place. The author has saved by this method two patients who would otherwise inevitably have perished, all other means having been used in vain.

*Spondylizema, or, Vertebral Sinking produced by Pott's Disease, as a new cause of pelvic alterations, compared with Spondylolisthesis, or Vertebral Gliding.* De Paul. (*L' Union Méd.*, January 25, 1877, No. 10).

The following are the author's conclusions:

1. Diseases of the lumbar and sacral vertebræ may induce two essentially distinct deformities, according as the caries affect the *body* or *arch* of the vertebra.

2. In the first, when the *body* is destroyed, which is the real support of the column, the latter sinks upon itself and becomes inclined. This inclination may produce such an extensive forward projection that it *covers* the superior strait and interferes with fœtal engagement in the canal. This lesion we have concluded to term *spondylizema*—vertebral sinking.

3. In the second deformity, when the vertebral arch is altered, which, by means of its apophyses and articular surfaces, maintains in position the column with the ligaments and muscles of this region, the spine, obeying the laws of gravity, *glides* forward into the pelvic cavity, and thus *obstructs*. To this lesion Kilian gave the name of *spondylolisthesis*—vertebral sliding.

4. In *spondylizema* the sacro-pubic diameter preserves its normal length. It may be even increased by reason of the diminution of the height of the base of the sacrum. But the *strait* to be passed by the fœtal head is pushed up higher. It is represented by a line which extends from the pubis to the body of one of the dorsal or lumbar vertebræ, brought nearer to the pubis by the forward inclination of the vertebral column.

5. In *spondylolisthesis* the sacro-pubic diameter is shortened by the interposition of the bodies of the lumbar vertebræ between the pubis and sacrum, in consequence of their gliding into the canal in advance of the sacrum.

6. The consequences of this last form of lesion are, as regards the pelvic cavity, more grave than in the former case. The two, when combined, may, as facts have abundantly demonstrated, lead to the most painful necessities of all operative midwifery.

